# SOCIAL INVISIBILITY OF ELDER ABUSE IN FAMILIES: A PHENOMENON IN PHILIPPINE SOCIETY

**DOI:** 10.1093/geroni/igae098.0953

**Published:** 2024-12-31

**Authors:** Carmelina Oyales, Victoria Apuan, Ronaldo Motilla

**Affiliations:** Chair; Co-Chair; Discussant

## Abstract

In a country where the prevailing culture puts a premium on respect and care for the elderly, elder abuse is an interesting phenomenon. The study focuses on elder abuse and the different forms of abuse occurring in Filipino communities. A qualitative study composed of individual interviews of 10 victims of elder abuse were undertaken. The aim is to explore the thoughts, feelings, sense of self, family dynamics and coping strategies of the abused. Further, data on the socio-cultural and economic risk and protective factors in the community and local government, the perspectives of 32 members of local government and social welfare institutions were gathered through focus group discussions in 5 localities. Narrative and content analysis yielded the following results: that elder abuse is a multifaceted and complex experience for the victims. Elder abuse victims suffered initial shock, denial, indignation, depression, and for some, learned helplessness. The most frequent type of abuse were emotional and psychological abuse, neglect, abandonment and financial exploitation. Poverty and ageism were an exacerbating factor. Actions to access local support mechanisms and interventions were absent or delayed due to the secrecy of the families regarding the phenomenon, as well as the lack of reporting and monitoring mechanisms on the community level.

Keywords: elder abuse, aging population, ageism, poverty, senior citizens human rights Aging Among Asians Interest Group Sponsored Symposium

